# Alveolar Soft Part Sarcoma of the Tongue: A Rare Tumor at an Unusual Location

**DOI:** 10.7759/cureus.40647

**Published:** 2023-06-19

**Authors:** Asim Qureshi, Asem Shalaby, Khamis Al Hasani, Eiman Al-Ajmi, Fizza Qureshi, Yahya Al Badaai, Juma Al Kasbi, Abdelhadi Shebl

**Affiliations:** 1 Histopathology, Sultan Qaboos University Hospital, Muscat, OMN; 2 Pahtology, Sultan Qaboos University, Muscat, OMN; 3 Faculty of Dentistry, Sultan Qaboos University hospital, Muscat, OMN; 4 Radiology, Sultan Qaboos University Hospital, Muscat, OMN; 5 Dentistry, Oman Dental College, Muscat, OMN; 6 Otolaryngology - Head and Neck Surgery, Sultan Qaboos university Hospital, Muscat, OMN; 7 Otolaryngology - Head and Neck Surgery, Sultan Qaboos Comprehensive Cancer Care Center and Research Center, Muscat, OMN; 8 Pathology, Sultan Qaboos University, Muscat, OMN

**Keywords:** tumor imaging, polypoidal tumor, excision, tongue, alveolar soft part sarcoma

## Abstract

Alveolar soft part sarcoma (ASPS) is a rare type of soft tissue sarcoma that typically affects adolescents and young adults, though it can occur at any age. We report a case of ASPS of the tongue, which is extremely rare at this location. The patient presented with a polypoidal lesion on the tongue, a biopsy of which showed granular and alveolar morphology. A definitive diagnosis was not rendered due to limited tissue. The case was discussed with the treating surgeon, and excision was recommended with clear margins. Excision of the lesion showed typical ASPS. A TFE-3 immunohistochemical stain was done, which showed strong immunoreactivity, thereby confirming the diagnosis of ASPS. This tumour is rare, and its presence in the tongue makes it extremely infrequent.

## Introduction

Alveolar soft part sarcoma (ASPS) is called "alveolar" because the tumour cells tend to grow in small spaces that resemble the air sacs (alveoli) in the lungs. ASPS usually originates in the muscles of the limbs, especially the legs, but can also occur in the trunk, head, and neck. It tends to grow slowly and may not cause symptoms for a long time, but it can eventually become quite large and invade nearby tissues and organs [1]. ASPS is a very rare sarcoma that accounts for 0.4% to 1% of all soft tissue sarcomas and typically occurs in adolescent and younger adult patients [2].

The cause of ASPS is unknown, and there are no known risk factors. A diagnosis is typically made through a combination of imaging studies, such as MRI or CT scans, and a tumour tissue biopsy. TFE-3 is an important immunohistochemical stain that stains the nuclei of tumour cells [3]. Despite its indolent behaviour, ASPS has a high potential for metastasis to the lungs, bones, liver, soft tissue, and brain. Only curative resection leads to a relatively better prognosis, whereas in cases where this is not possible, the prognosis is often poor, with a median survival of around 40 months [4].

## Case presentation

**Figure 1:**
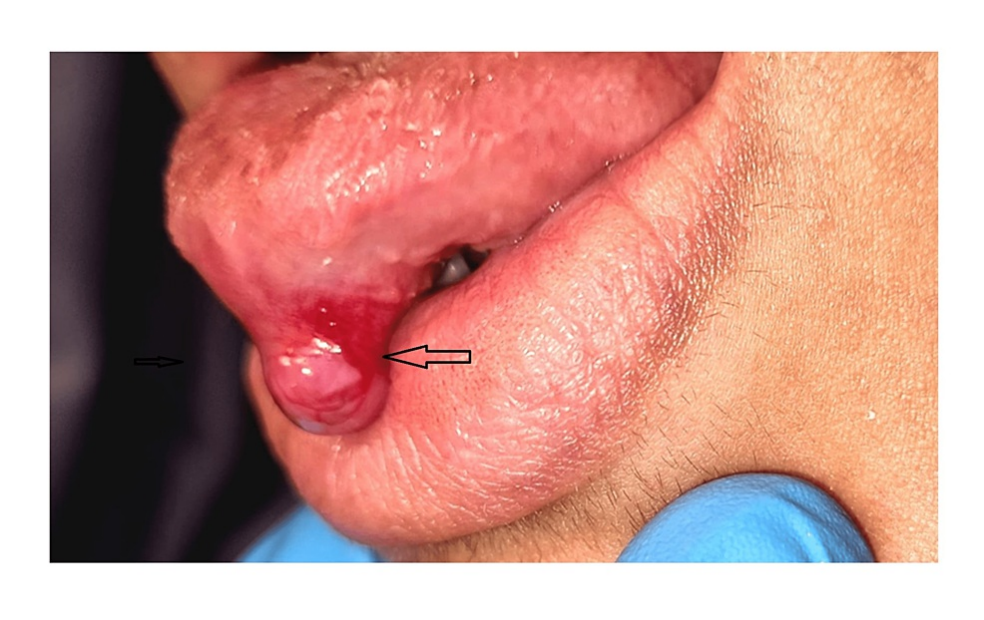
Clinical picture Photograph of the patient showing a polypoidal tumor at the ventral surface of the tongue.

A 22-year-old female with known sickle cell disease presented with a polypoidal lesion on the ventral surface of the tongue (Figure 1).

**Figure 2:**
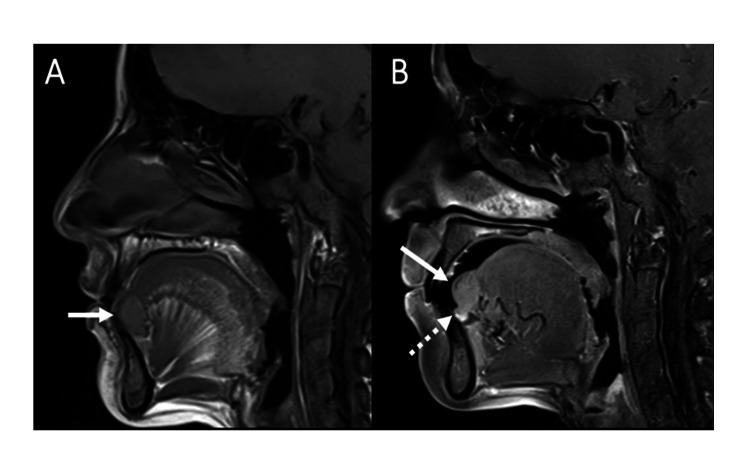
Radiology image MRI of the tongue: (A) Sagittal T1-weighted image shows the lesion at the volar aspect of the anterior third of the oral tongue (arrow), with the lesion invading the intrinsic muscles of the tongue and inseparable from the most anterior aspect of the genioglossus muscle. The lesion is mildly T1 hyperintense relative to the muscles. (B) Gadolinium-enhanced sagittal T1-weighted image shows enhancement of the lesion. This image demonstrates the bilobed appearance of the lesion with a main component superiorly (solid arrow) and a small inferior exophytic component (dashed arrow).

Magnetic resonance imaging (MRI) of the tongue was performed and demonstrated the presence of a bilobed lesion arising from the volar aspect of the tongue at the midline, invading the intrinsic muscles of the tongue and the genioglossus muscle (Figure 2).

**Figure 3:**
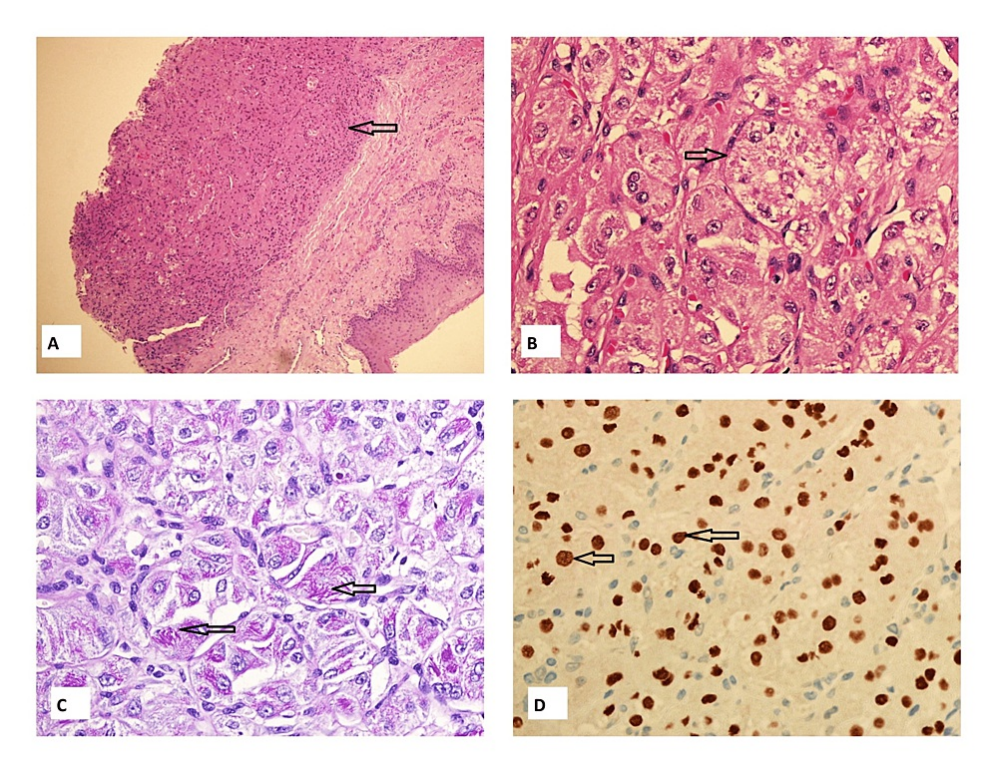
Histology images (A) Biopsy of the lesion H and E stained section at 4× magnification. shows tumor cells arranged in nets showing distinct granular pattern. (B) Hand E stained slide at 40× magnification showing alveolar pattern. (C) PAS with diastase showing intracytoplasmic crystalline material. (D) TFE-3 IHC stain showing nuclear positivity in tumor cells.

The lesion measured 13 mm × 12 mm × 17 mm. It showed mild T1 hyperintensity relative to the normal muscles and T2 hyperintensity, which was enhancing. Prominent vessels were noted adjacent to the lesion, but there were no flow voids within the lesion. There was no cervical adenopathy by imaging criteria. A biopsy was done, which showed a lesion with the morphology of a granular cell lesion. Immunohistochemistry (IHC) was performed (Figure 3A). A S100 IHC stain was done on the tissue, which was negative. CD 68 showed patchy, variable reactivity. The rest of the immunohistochemical workup, including smooth muscle, skeletal muscle, and neural and epithelial origin stains, was negative. A diagnosis of a non-neural ganglion cell tumour was rendered at the time, keeping in view the cells' granular appearance and the tumour's location. The case was discussed with the treating physician, and it was mutually decided that an excision with clear margins is required.

The lesion was excised with clear margins, and the specimen was sent to the pathology lab. The frozen section for margins was not performed. Sections from the periphery of the lesion were similar to the biopsy; however, the central portion of the lesion showed a distinct alveolar pattern (Figure 3B). The cells were large, with abundant cytoplasm and intracytoplasmic crystals highlighted by the PAS diastase stain (Figure 3C). The results of the IHC panel performed on the excised tumour were the same as those of the biopsy. Owing to the alveolar pattern, a provisional diagnosis of ASPS was considered, and the slides were sent for TFE-3 staining (Figure 3D).

These showed strong nuclear immunoreactivity, therefore confirming the diagnosis of ASPS. The case was discussed in a multidisciplinary team meeting. Owing to one close margin, excision versus radiation to the site of excised tumor were offered. It was decided in the MDT to go for resection for clear margins, as 1cm or more was considered a safe margin. The re-resected specimen showed clear margins and the patient was referred to radiation therapy for evaluation.

## Discussion

The incidence of ASPS in the general population is extremely rare [1]. The tongue is an extremely rare site for the development of this tumour. A typical pattern owing to necrosis in individual nests, thereby creating a central area of clearing with tumour cells set in the periphery, giving an alveolar look [2].

The majority of the IHC markers are negative except for focal staining for CD68. TFE-3 is a transcription factor that is a strong and specific stain for ASPS. It shows strong nuclear positivity [3,4]. Radiologically, ASPS tumours in the head and neck have similar features to those that occur elsewhere in the body. These tumours are described as being isointense to mildly T1 hyperintense relative to the normal muscles, as shown in our patient. They are hyperintense on T2-weighted images and show avid enhancement. Internal flow voids and prominent peritumoral vessels are classic imaging features in ASPS [5-7].

Treatment usually involves surgical removal of the tumour, sometimes with the addition of radiation therapy or chemotherapy. Because ASPS is a rare and slow-growing cancer, it is important to receive treatment from a specialist who has experience managing this condition.

ASPS is a rare type of sarcoma that usually affects adolescents and young adults. It typically originates in the soft tissues of the body, such as muscles and tendons, and can spread to other areas of the body, including the lungs, brain, and bones [8,9]. The exact cause of ASPS is not yet known, and it is currently classified as a type of soft tissue sarcoma. Common symptoms of ASPS include swelling, pain, and tenderness in the affected area. The diagnosis usually involves a combination of imaging tests, such as MRI or CT scans, and a biopsy of the affected tissue [10,11].

A scoping review done in Iran by Akinyamoju et al. concluded that the tongue is more commonly affected in females. Also, the base of the tongue was the most common location affected, while surgical management was mostly used for treatment, and cases managed by surgery alone were free of disease at ≤5 years of follow-up [12].

Lucas et al. reported that surgical extirpation and microvascular reconstruction were successful, and the patient remained disease-free four years post-operatively [13]. The treatment offered to our patient was also surgical with curative intent.

Alegria Landa et al. reported ASPS of the tongue as a highly vascularized tumour with small vascular spaces separating nests of cells characterized by chromosome rearrangement der(17)t(X:17)(p11:q25) that results in the ASPL-TFE3 translocation [14]. Our tumour also showed a vascular pattern in the centre of the lesion and strong TFE-3 positivity.

Gong et al. report that ASPS rarely occurs in the head and neck, and it occurs more rarely in the tongue. The prognostic factors of ASPS are related to the age of onset, the size of the primary lesion, whether the surgical resection is complete, and whether metastasis has occurred. Due to the short course of ASPS that occurs in the tongue and its smaller size, the prognosis is better after complete resection [15].

Hsu et al. reported a 1.5-2.0 cm clinically safe margin under general anaesthesia, and the surgical defect was repaired by primary closure. The histopathological diagnosis was again ASPS, with a microscopic safe margin of 0.8 cm. The patient revealed recovered swallowing and phonetic function at one-month postoperative follow-up [16]. In our case, a safe margin of 1 cm was considered safe.

Carson et al. believe that early age and mucosa site are atypical features of this case and could possibly have a different histogenesis from the typical alveolar soft part sarcoma found in a younger population [17]. Our patient is 22 years old; therefore, resection of the margins was considered.

Zichao Tan et al., in a single-centre study, show ASPS exhibits a higher occurrence in the head and neck in children. ASPS originating from the glossopharyngeal region tends to have a lower metastasis rate [18]. Our patient was extensively imaged to look for metastatic disease.

Anbarasi et al. reported a difficult case of ASPS that resembled a hamartomatous granular cell tumour (GCT). They reported this was a challenging case, and the confirmatory diagnosis was largely based on IHC staining for TFE-3. Also, they stated The prognostic parameters of ASPS include age at diagnosis, tumour size, and the presence of metastasis [19]. This case is quite similar to ours, as the punch biopsy done in the first instance in this published case also simulated a granular cell tumour.

Raghunandan et al. reported a case of ASPS in which they embarked on performing a wide surgical resection to reduce the risk of local recurrence. Pre-operative staging proves vital due to the frequency of metastases at presentation and the relatively poor prognosis of ASPS. Long-term post-operative follow-up is mandatory for all patients with this deceptively indolent and highly malignant neoplasm [20]. As noted earlier, if the team decides on surgery with curative intent, the case should be extensively worked up for metastatic disease.

Kinger et al. reported ASPS, one of the very few cases of lingual ASPS reported from India. ASPS should be considered a differential diagnosis of lingual soft tissue mass in young children due to their tendency for early metastasis. Histopathological examination and special stains help in confirming the diagnosis of ASPS [21]. By far the majority of the cases reported in the head and neck area are in young people.

ASPS accounts for less than 1% of all sarcomas. More frequently encountered within the lower limbs, the authors present a 24-year-old male with ASPS presenting as an asymptomatic swelling of the lateral tongue. At 12 months post-wide local excision of the lesion, the patient remains well with no evidence of local or regional recurrence. Histological and immunohistochemical features are diagnostic of ASPS. Whilst rare, head and neck surgeons should be aware of ASPS as a potential cause of slow-growing lesions, as early surgical resection is vital in view of the propensity for metastatic spread [22].

Treatment for ASPS typically involves a combination of surgery, radiation therapy, and chemotherapy. However, ASPS is generally resistant to chemotherapy, so treatment can be challenging. The prognosis for ASPS can vary depending on several factors, such as the size and location of the tumour as well as the stage of the disease at the time of diagnosis [22].

## Conclusions

To the best of our knowledge, we are presenting the first case of ASPS of the tongue from the region. A small biopsy of the lesion may be deceptive, as it was in our case. Therefore, it is mandatory to be in contact with the treating surgeon regarding acquiring a bigger tissue piece. The key to successful treatment is clinicopathological correlation and good communication between diagnostic and clinical services.
